# Prevalence of Undiagnosed Iron Deficiency Anemia and Associated Factors Among Female Undergraduate Medical Students in Makkah, Saudi Arabia

**DOI:** 10.7759/cureus.50046

**Published:** 2023-12-06

**Authors:** Hibah A Almasmoum, Mohammad Shahid Iqbal, Abeer Aljaadi, Kholoud Ghafouri, Ahmed H Qasem, Wedad Azhar, Alaa Qadhi, Amr J Halawani, Amal Ezzat Abd El-Lateef, Ashwaq Alharthi, Athar Khoja

**Affiliations:** 1 Department of Clinical Laboratory Sciences, Faculty of Applied Medical Sciences, Umm Al-Qura University, Makkah, SAU; 2 Department of Pathology, Medicine Program, Batterjee Medical College, Jeddah, SAU; 3 Department of Clinical Nutrition, Faculty of Applied Medical Sciences, Umm Al-Qura University, Makkah, SAU; 4 Department of Clinical Pathology, Faculty of Medicine, Tanta University, Tanta, EGY

**Keywords:** saudi arabia, dietary intake, female medical students, prevalence, iron deficiency anemia

## Abstract

Introduction: Iron deficiency anemia is the most common type of anemia according to the World Health Organization. Females are more likely to be affected than males. There are several factors causing iron deficiency anemia, such as increased loss of iron, decreased intake of iron, and increased utilization of iron. There are limited research studies evaluating the risk factors that cause anemia among female medical students in Saudi Arabia. For this, the study aimed to identify the prevalence of undiagnosed iron deficiency anemia (IDA) among young female university students and to identify if there is any correlation between IDA and several factors including dietary habits, psychological stress, anxiety status, and body mass index.

Materials and methods: A cross-sectional observational study was performed between October 2022 and December 2022 with a sample of 100 healthy female students aged between 19 and 23 years, who were studying at the medical colleges of Umm Al-Qura University, Saudi Arabia. Blood samples were collected to perform complete blood count and iron profile tests. Also, a survey was done to find correlation between iron deficiency anemia and dietary intake, drinks, stress, anxiety, and body mass index (BMI). Independent t-test or Mann-Whitney U test were used to compare values for non-anemic with anemic participants, and linear regression tests were used to analyze differences between non-anemic and anemic participants' dietary intake factors and stress and anxiety scores. The level of significance was set at p<0.05.

Results: The findings represent an overall prevalence of iron deficiency anemia in female medical students based on the lab finding results. Thus, students were divided into two following groups: anemic (13%) with hemoglobin (Hb) concentration <12 g/dL and non-anemic (84%) with Hb concentration ≥12 g/dL. When comparing the two groups, results showed significant differences in the majority of RBC indices (hematocrit {HCT}, mean corpuscular volume {MCV}, mean corpuscular hemoglobin {MCH}, red cell distribution width {RDW}) and iron profiles, p-value was <0.05. On evaluation of the different types of food consumption, the mean iron intake was around 7 mg/day, and in 65% of the participants, it was below the estimated average requirement (EAR) of iron (8.1 mg/dL). The perceived stress scale (PSS) shows that 63% of the participants experienced moderate stress and 58% reported severe anxiety by the generalized anxiety disorder (GAD) scale. In multiple linear regression, iron was positively associated with hemoglobin concentrations, whereas stress scores were negatively associated with hemoglobin concentrations.

Conclusion: There is a relatively low prevalence of anemia and most of it was found of the microcytic type, indicating that this condition is a common health issue among local female college students. There was no independent association between stress or anxiety and hemoglobin levels.

## Introduction

Iron deficiency anemia (IDA) is the most common cause of nutritional anemia worldwide followed by other nutritional deficiencies, such as folate, vitamin B12, vitamin B6, and vitamin C [1,2]. According to the World Health Organization (WHO), over one-fourth of the world’s population is anemic, the major cause being iron deficiency [1,3,4]. An imbalance between the physiological demand and dietary intake of iron results in its deficiency. There are various causative factors for IDA, some are modifiable whereas others are non-modifiable, many of them coexist and result in IDA [2].

A hemoglobin concentration below 12 g/dL in non-pregnant females and below 13 g/dL in males is considered anemia as defined by WHO. IDA is more common in developing countries. However, its burden is quite significant in developed countries including Gulf countries, with a high frequency of anemia in females between the ages of 17 and 24 years [4]. Saudi Arabia reports a prevalence of IDA between 20.0% and 39.9% among susceptible groups, such as preschool children and females in the reproductive age group [4].

Iron deficiency anemia (IDA) usually results from low dietary intake, blood loss resulting in loss of iron, defective iron absorption, and chronic diseases, such as end-stage kidney disease and inflammation [5]. Strict vegetarian diets, chronic blood loss resulting from heavy menstrual losses, blood loss from gastrointestinal tract due to stomach ulcers, angiodysplasia and malignancy, and regular blood donation are the other significant causes of IDA [6]. Studies have shown evidence that IDA is associated with psychosocial consequences, including adverse psychomotor function, reduced work capacity, and psychiatric morbidities, such as anxiety, depression, bipolar disorders, sleep disorders, and restless legs syndrome [7,8]. Whereas, other authors have reported no association between IDA and psychotic disorders [9,10].

IDA is clinically silent and significant clinical features appear only with moderate to severe anemia. Tiredness, fatigue, headache, dizziness dry mouth, cheilitis, atrophic glossitis, Plummer-Vinson syndrome, and restless legs are the usual symptoms and are observed in longstanding deficiency [7,8]. The laboratory tests used to confirm IDA include serum ferritin, total iron binding capacity (TIBC), and transferrin saturation. A low serum ferritin level is a good indicator of iron deficiency, reflecting exhausted stores. Levels of <30 mg/L are the accepted threshold that identifies mild cases; in the presence of anemia, ferritin levels are usually lower (<10-12 mg/L). Reduction of mean corpuscular hemoglobin (MCH) and mean corpuscular volume (MCV) occur relatively late because of the erythrocyte lifespan [7]. Therefore, this study aimed to identify the prevalence of undiagnosed IDA among young female university students and to identify if there is any correlation between IDA and several factors including dietary habits, psychological stress, anxiety status, and body mass index.

## Materials and methods

Subjects

A cross-sectional observational study was performed between October 2022 and December 2022 with a sample of 100 healthy undergraduate female students aged between 19 and 23 years. The participants were studying pharmacy, nursing, and applied medical sciences (clinical laboratory sciences, physical therapy, clinical nutrition) at the medical colleges of Umm Al-Qura University, Saudi Arabia.

Regarding the sample size calculations, a sample size of n=63 was estimated using G*Power 3.1 (Düsseldorf, Germany: Heinrich-Heine-Universität Düsseldorf) to detect a medium effect size in the linear regression models (f2=0.15) given a power of 0.85 and including a maximum of 10 predictors with alpha level of 0.05. For the sampling technique, we used convenience sampling as we advertised for the study and participants who were interested in the study and met the inclusion criteria were recruited.

The study sample excluded students who reported being diagnosed with any type of anemia, pregnancy, breastfeeding, or students who took iron supplements. All participants were provided with written informed consent acknowledging the purpose of the investigation and were assured of the confidentiality of the results.

Data collection

Primary Survey

This survey was developed by the SurveyMonkey online platform and was provided in the Arabic language, consisted of 35 questions, and divided into three sections. The first section included the participant's consent for this study sociodemographic data (age, weight, marital status, height, medical specialty, and their level), daily exercise, medical history (e.g., taking iron supplements during the past three months, health problems need medication), students on iron supplements, pregnant, breastfeeding, and any type of anemic disorders were excluded from further participation.

The second section included a food frequency questionnaire (FFQ). A detailed inquiry was made regarding the students’ drink habits (e.g., favorite drinks, consuming coffee, and tea either immediately after the meal or half an hour later), dietary intake of various foods (e.g., 100 g red meat, 100 g poultry, 50 g cheese, 50 g fish, 100 g fresh fruits, 50 g egg, 250 g dairy products, 100 g grains, 100 g vegetables, 10 g fat, 30 g nuts, and seeds, 35 g brown flour bakery) [11,12]. The iron intake was estimated by multiplying the daily number of servings by the quantity of iron in one serving. The total daily iron intake was the sum of iron in all the food items consumed per day.

The third section of the survey included the Arabic translation of perceived stress scale (PSS) and generalized anxiety disorder (GAD) to assess stress and anxiety among these students [13,14].

Blood Sample Collection

Venous blood samples were collected in 6-7 mL ethylenediaminetetraacetate (EDTA)-coated and plain tubes for each participant simultaneously along with the survey. These samples were immediately sent for analysis to a private laboratory Wateen Algalb in Makkah city accredited by the Saudi Central Board for Accreditation of Healthcare Institutions (CBAHI). Complete blood count (CBC) and serum iron profile were performed for each sample.

CBC and Iron Profiles

Complete blood count (CBC) included RBC, hemoglobin (Hb) concentration, and RBC indices, such as mean corpuscular hemoglobin concentration (MCHC), mean corpuscular hemoglobin (MCH), mean corpuscular volume (MCV), and red cell distribution width (RDW). WBC counts and their differential count (absolute and percentage) and platelets counts were performed using a Beckman Coulter DxH 520 machine (Brea, CA: Beckman Coulter, Inc.).

Serum samples were used for iron profile using a UDILIPSE clinical chemistry analyzer (Dammam, SA: United Diagnostic Industry) and MAGLUMI 2000 Plus (Shenzhen, China: Snibe Co. Ltd.) automatic immunoassay analyzer. The following range was used as normal and any values below that range were considered deficient. The normal range for iron profile taken was as follows - serum iron (SI): 37-170 µg/dL, serum ferritin (SF): 30-232 ng/mL, and total iron binding capacity (TIBC): 250-450 µg/dL. The defining criterion for anemic participants was Hb<12 g/dL, SI<37 µg/dL, SF<30 ng/mL, TIBC>450 µg/dL. For participants with iron deficient but non-anemic defining criterion was Hb>12 g/dL and SF<30 ng/mL.

Statistical analysis

Data are presented as means±standard deviation (SD), median (25th, 75th percentiles), or n (%) as appropriate. Independent t-test and Mann-Whitney U tests were used to compare continuous variables between two groups for normally and non-normally distributed variables, respectively. Data were compared between three groups using analysis of variance (ANOVA), or its non-parametric equivalent Kruskal-Wallis if assumptions were not met. Multiple linear regression was used to assess the association between continuous outcomes (Hb and iron status) with iron intake, the timing of coffee and tea consumption, stress scores, anxiety scores, menstruation duration, and the body mass index (BMI). Due to skewed residuals in some models, hemoglobin was cubic transformed, and ferritin was log-transformed. Significance was indicated by two-sided p-values<0.05. Data were analyzed with Stata software version SE/14.2 for Mac (College Station, TX: Stata Corp.).

## Results

Participants characteristics

The current study included 100 healthy female students from undergraduate medical disciplines with a mean age of 20.7±0.8 years. Only 4% of participants were married but neither pregnant nor breastfeeding. The majority of students were from college applied medical science 59% and others from nursing, pharmacy, and medicine with 30%, 9% and 2%, respectively. Based on their BMI, 27% were overweight/obese, 24% were underweight, and rest were normal. On evaluation of the different types of food consumption, the mean iron intake was around 7 mg/day, and in 65% of the participants, it was below the estimated average requirement (EAR) for iron (8.1 mg/day). The PSS shows that 63% of the participants experienced moderate stress and 58% reported severe anxiety on GAD scale. About 48% of participants in this study preferred coffee of all kinds, and the remaining preferred tea and soft drinks about 20% and 13%, respectively. Only 19% of participants did not prefer any type of caffeine drink. Furthermore, about 10% of participants with normal iron status drink always caffeine after meal or within half an hour. The majority of the students were occasionally drinking caffeine. About 82.8% of students had moderate menstruation, while 9.2% and 8% had either light or heavy menstruation, respectively. While the duration of menstruation, 23% had less than five days, 69% had five to seven days, and only 8% had above seven days of menstruation. The participants’ characteristics are shown in Table 1.

**Table 1:** Sociodemographic and lifestyle characteristics of young Saudi females (n=100). Data are presented as mean±SD and n (%). EAR: estimated average requirement

Characteristics		Value
Age (years)		20.7±0.8
Marital status	Single	96 (96%)
	Married	4 (4%)
BMI (kg/m^2^)		22.1±4.7
Underweight (BMI<18.5)		24 (24%)
Healthy (BMI=18.5-24.9)		49 (49%)
Overweight/obesity (BMI≥25)		27 (27%)
College	Applied Medical Science	59 (59%)
	Nursing	9 (9%)
	Pharmacy	30 (30%)
	Medicine	2 (2%)
Iron intake (mg/dL)		6.64±1.93
< EAR (8.1 mg/day)		65 (65%)
Perceived stress score		22.6±6.0
Low		6 (6%)
Moderate		63 (63%)
High		31 (31%)
Perceived anxiety score		15.4±4.8
Mild		3 (3%)
Moderate		39 (39%)
Severe		58 (58%)
Daily workout	Never	36 (36%)
	Half an hour	51 (51%)
	One hour	10 (10%)
	Two hours	2 (2%)
	Three hours	1 (1%)

Factors associated with hemoglobin concentrations and iron biomarkers

Hematological parameters showed that 13% of the females were anemic with a mean hemoglobin of 10.88±0.76. The RBC count, MCV, MCH, MCHC, and hematocrit (HCT) were significantly lower (p<0.05) among the anemic group compared to the non-anemic group (Table 2). Serum iron levels were below the normal range among the anemic group with a median of 31 (28, 36) μg/dL and were significantly lower than the non-anemic group with a median of 74 (54, 103) μg/dL (p=0.000). TIBC was significantly higher among the anemic group with a median of 476 (444, 493) μg/dL and the non-anemic group with a median of 415 (359, 450) μg/dL (p=0.004). Serum ferritin was also significantly lower among the anemic group with a median of 4 (3.3, 6.6) ng/mL compared to the non-anemic group with a median of 16.6 (8.1, 33.7) ng/mL (p=0.000) (Table 2). However, there was no significant difference in age and BMI in anemic vs. non-anemic participants (data not shown).

**Table 2:** Biochemical characteristics of young Saudi females by anemia status. Data are presented as mean±SD or median (25th, 75th percentile). Independent t-test or Mann-Whitney U test were used to compare values of non-anemic with anemic participants. HCT: hematocrit; MCV: mean corpuscular volume; MCH: mean corpuscular hemoglobin; MCHC: mean corpuscular hemoglobin concentration; RDW: red cell distribution width; PLT: platelet; MPV: mean platelet volume; TIBC: total iron binding capacity

Biomarker	Total (n=100)	Anemic (n=13)	Non-anemic (n=87)	p-Value
Hemoglobin	13.01±1.11	10.88±0.76	13.33±0.74	0.000
RBC	4.61±0.43	4.27±0.36	4.66±0.42	0.002
HCT	37.95±2.82	33.73±1.92	38.73±1.97	0.000
MCV	82.95±6.09	76.91±4.74	83.85±5.76	0.000
MCH	28.46±2.53	25.54±1.80	28.89±2.33	0.000
MCHC	34.29±0.76	33.36±0.46	34.43±0.70	0.000
RDW	14.59±1.32	16.04±1.29	14.37±1.19	0.000
PLT	311.05±60.98	343 (333, 429)	311 (266, 345)	0.023
MPV	9.29±0.88	9.32±0.94	9.28±0.87	0.561
Iron	69 (47, 94)	31 (28, 36)	74 (54, 103)	0.000
TIBC	410.92±73.99	476 (444, 493)	415 (359, 450)	0.004
Ferritin	13.25 (6.55, 29.3)	4 (3.3, 6.6)	16.6 (8.1, 33.7)	0.000

On comparison of groups with sufficient and insufficient iron intake serum iron values were lower among the insufficient intake group (66.8±30.435.5 μg/dL) than the participants with appropriate EAR for iron (83.8±36.3 μg/dL) with a significant p-value of 0.014. Hemoglobin level and serum ferritin were also lower in the insufficient intake group and TIBC was higher with a mean of 412±72 µg/dL (Table 3). The hemoglobin level and iron profile showed no significant difference among the participants with low, moderate, or high stress as well as anxiety levels (Table 3).

**Table 3:** Hemoglobin and iron biomarkers by iron intake, stress status, and anxiety levels. Data are presented as mean±SD or median (25th, 75th percentile). Data were compared between categories using two-sample t-test, ANOVA, or their non-parametric equivalent: Mann-Whitney U test or Kruskal-Wallis, respectively. TIBC: total iron binding capacity

Variables		Biomarkers			
		Hemoglobin	Serum iron	Ferritin	TIBC
Iron intake	Insufficient (<8.1 mg/day), n=65	13.0 (12.6, 13.7)	66.8±30.4	12.6 (5.80, 27.6)	412±72
	Sufficient (≥8.1 mg/day), n=35	13.1 (12.7, 14.1)	83.8±36.3	21.3 (7.70, 40.8)	409±78
p-Value		0.136	0.014	0.103	0.828
Perceived stress levels	Low, n=6	12.95 (12.8, 14)	66.5 (54, 73)	12.55 (4.7, 16.4)	421 (380, 435)
	Moderate, n=63	13.1 (12.6, 13.7)	71 (46, 93)	17.8 (7.4, 32.1)	415 (366, 473)
	High, n=31	13.1 (12.5, 13.9)	75 (47, 105)	12.6 (4.9, 28.6)	433 (359, 454)
p-Value		0.990	0.356	0.281	0.957
Perceived anxiety levels	Mild, n=3	13 (12.9, 14.0)	73 (54, 74)	14.3 (4.7, 16.4)	425 (417, 445)
	Moderate, n=39	13.1 (12.6, 13.6)	61 (41, 78)	11.1 (6.5, 28.4)	421 (368, 462)
	Severe, n=58	13.1 (12.6, 13.9)	76 (47, 105)	15.25 (7.0, 33.7)	415.5 (359, 469)
p-Value		0.842	0.183	0.765	0.926

In multiple linear regression, iron was positively associated with hemoglobin concentrations, whereas stress scores were negatively associated with hemoglobin concentrations. Anxiety and ferritin were not associated with hemoglobin concentrations after adjusting for BMI, menstruation, iron intake, and timing of drinking coffee and tea. Serum iron concentrations were not associated with iron intake but were negatively associated with BMI after adjusting for menstruation and the timing of drinking coffee and tea. None of the factors were associated with ferritin in the multiple linear regression model and the model only explained 8% of the variation in ferritin concentrations (Table 4).

**Table 4:** Factors associated with hemoglobin concentrations and iron biomarkers. Multiple linear regression (n=90) was used to assess the association between the outcome and iron intake, timings of the coffee and tea consumption, stress scores, anxiety scores, mensuration intensity, and duration, adjusting for BMI. *Due to skewed residuals in the model, hemoglobin was cubically transformed and ferritin was log-transformed.

Outcome	Biomarkers (95% CI)	p-Value
Hemoglobin concentrations^* ^(model R^2^ = 0.47)		
Serum iron	8.76 (5.91, 11.6)	<0.001
Ferritin	3.77 (-0.60, 8.14)	0.090
BMI	14.9 (-8.74, 38.5)	0.213
Iron intake	-26.0 (-84.3, 32.3)	0.377
Drink timings with/after food (30 min) (reference: always)		
Sometimes	152 (-90.9, 394)	0.217
Never	46.7 (-271, 364)	0.770
Stress scores	-19.67 (-39.29, -0.05)	0.049
Anxiety scores	9.05 (-18.0, 36.1)	0.507
Menstruation intensity (reference: light)		
Moderate	-203 (-592, 187)	0.303
Heavy	-466 (-953, 21.6)	0.061
Menstruation duration (reference: <5 days) > 7 days		
5-7 days	198 (-51.0, 447)	0.117
> 7 days	-37.8 (-350, 275)	0.811
Serum iron concentrations (model R^2^ = 0.23)		
BMI	-1.94 (-3.11, -0.75)	0.002
Iron intake	2.33 (-1.45, 6.11)	0.224
Drink timings with/after food (30 min) (reference: always)		
Sometimes	11.1 (-8.25, 30.4)	0.257
Never	4.97 (-24.1, 14.1)	0.606
Stress scores	0.074 (-1.65, 1.80)	0.933
Anxiety scores	1.70 (-0.386, 3.79)	0.109
Menstruation intensity (reference: light)		
Moderate	18.8 (-1.23, 38.8)	0.065
Heavy	11.8 (-15.4, 39.0)	0.391
Menstruation duration (reference: <5 days)		
5-7 days	6.12 (-9.92, 22.1)	0.450
> 7 days	10.86 (-17.98, 39.7)	0.456
Ferritin concentrations^*^ (model R^2^ = 0.08)		
BMI	0.004 (-0.041, 0.049)	0.871
Iron intake	-0.007 (-0.142, 0.129)	0.923
Drink timings with/after food (30 min) (reference: always)		
Sometimes	0.246 (-0.425, 0.918)	0.467
Never	-0.156 (-0.867, 0.555)	0.664
Stress scores	-0.040 (-0.085, 0.005)	0.082
Anxiety scores	0.041 (-0.020, 0.102)	0.181
Menstruation intensity (reference: light)		
Moderate	0.185 (-0.524, 0.894)	0.605
Heavy	-0.114 (-1.193, 0.965)	0.834
Menstruation duration (reference: <5 days)		
5-7 days	0.258 (-0.276, 0.792)	0.339
> 7 days	0.178 (-0.505, 0.861)	0.605

## Discussion

In the present study, we aimed to identify the prevalence of undiagnosed iron deficiency anemia among young female university students and to identify if there is any association between IDA and dietary habits, psychological stress, anxiety status, and body mass index.

Iron deficiency is the most common micronutrient deficiency worldwide with iron deficiency anemia being the most common nutritional anemia, especially among susceptible groups, such as preschool children, females in reproductive age group, and pregnant and lactating females [15]. The anemia prevalence in our cohort was 13%. The prevalence of anemia in other reports from university students across Saudi Arabia is between 29.3% and 67.35% [1,15-18]. Similar studies from outside Saudi Arabia have reported a prevalence of 36.25-54% [19-21]. Another study done within Saudi Arabia in females of reproductive age group also reported a high prevalence of 40% [22]. Whereas, a study in a similar age group from Iran reported a low incidence of anemia at 3.8% although there was a high percentage (40.9%) of iron-deficient non-anemia participants [23].

Among the anemic group in this study, there was a low mean serum iron, low MCV, and low MCH with elevated mean RDW indicating it to be iron deficiency anemia. The mean iron intake of all students was 6.64±1.93 mg/day with 65% of students taking low iron (<8.1 mg/day) compared to EAR. Among the anemic students, the mean hemoglobin and mean HCT were 10.88±0.76 and 33.73±1.92, respectively. A study conducted in Riyadh on female adults found that their average iron intake (10.22±8 mg/day) fell short of the dietary reference intake (DRI) recommendation [24]. Females' mean hemoglobin (13.68±0.83 g/dL) and mean HCT (38.9±3.2%) levels were below the cutoff values for diagnosing iron deficiency anemia. According to hemoglobin and HCT levels, 21.6% and 20.6% of female participants were anemic, respectively. In terms of iron intake, 95.1% of female participants fell below the recommended daily intake [24].

Loss of iron during menstruation appears to be the most common cause of IDA in this study. However, further investigations are needed to rule out other conditions causing anemia, such as celiac disease. Anemia caused by menstrual blood loss persists in young females into their late adolescent years. Consequently, they may continue to be anemic or develop new anemia because of the increased micronutrient demands associated with menstruation, pregnancy, and lactation [25]. Hence, dietary improvement of iron intake and regular monitoring of anemia in female students will help to bring down the anemia prevalence among females of reproductive age group. Teenagers in Saudi Arabia have a high incidence of iron deficiency anemia, which is caused by an inadequate intake of iron and a lack of knowledge about the importance of maintaining a healthy diet that includes a variety of foods that are high in iron [26].

The mean BMI for our cohort was 22.1±4.7 and the majority of the students (49%) were in the healthy BMI range and 27% of students were obese. A study from Peru among students reported a mean BMI of 24.01±3.64 with 26.1% of female students as overweight and 12.4% having anemia which is similar to our findings [25]. Another study among students from Bangladesh reported an anemia prevalence of 63.3% and majority of the female students had normal BMI with 18.1% in the overweight category, similar to our findings [27].

In the present study, following food frequency survey findings and based on multiple linear regression, there was an association between iron intake and serum iron level but not between hemoglobin and serum ferritin levels with iron intake (Table 3). However, another study from Yemen revealed a correlation between the lifestyle habits of university students, such as irregular consumption of meat, fish, and chicken, drinking tea, smoking, and the development of anemia. In addition, the study found that students' consumption of soft drinks or "colas" and coffee has a significant negative impact on their health and plays a significant role in the development of anemia [20]. The contrasting finding in our cohort might be due to small sample size. Along with age, physiological state, socioeconomic status, and nutritional status are also important risk factors for IDA. In a study conducted in Korea, the frequency of IDA in the general population was shown to be two to four times greater (11% vs. 3%) among underweight individuals compared to those with a normal BMI. In the same study, IDA was found in 7.4% of participants who had an inadequate iron intake and in 2.9% of those who had an adequate iron intake. However, IDA was not linked to either protein or total calorie consumption [28]. A low socioeconomic status, being underweight, and eating a diet low in iron or vitamin C were all linked to IDA [28].

The perceived stress survey showed that the majority of students (65%) perceive a moderate level of stress whereas 31% of students were severely stressed. Also, the anxiety scale showed that majority of students (58%) have severe anxiety. However, the data did not show a correlation between hemoglobin and iron biomarkers in stress and anxiety categories. There are reports in the literature describing the association between anemia and depression [29], but there is no independent association between depressive and/or anxiety disorders and hemoglobin levels or anemia status [10,30].

In our cohort, based on multiple linear regression, serum iron level was positively associated with hemoglobin concentrations, whereas stress scores were negatively associated with hemoglobin concentrations. Anxiety and ferritin were not associated with hemoglobin concentrations after adjusting for BMI, menstruation, iron intake, and timing of drinking coffee and tea. Iron is involved in numerous neurological processes, and iron deficiency is linked to anxiety, depression, and developmental issues [8].

Furthermore, there was a negative association between iron concentration and BMI. In contrast, a study done in rats demonstrated that psychological stress could decrease iron absorption in rats, which may be related to regulation of iron transporter gene expression [31]. There is a report that mentions children who are iron deficient have increased anxiety and/or sadness, as well as social and attentional issues [32].

Our study has some strengths, as we targeted women of childbearing age who are vulnerable to anemia and iron deficiency. Multiple biomarkers of anemia and iron status have been used and we collected both biochemical, dietary, and anthropometric measurements with measures of anxiety and stress. However, our study had several limitations. Convenience sampling and targeting only medical students could have resulted in selection bias as the more interested in research volunteered to participate. Participants were mainly young and only from a single university in the Western region of Saudi Arabia, which limits the generalizability of our findings to all females in the region or in Saudi Arabia. Self-reported data could result in misreporting by either over or under-reporting due to recall bias or social desirability bias. Moreover, no causal associations could be established between the influencing factors and outcomes due to the cross-sectional nature of the study.

## Conclusions

Despite the widespread knowledge about iron deficiency anemia, the prevalence of undiagnosed cases is quite high among female medical students, especially iron deficiency without anemia. It also shows a strong relationship between serum iron levels and iron intake. This emphasizes the need for health education and nutritional initiatives regarding ID and IDA among this female age group and also regular screening of this age group for IDA. Thus, the health policies regarding health education and screening of anemia can be targeted toward these susceptible groups. There was no significant association between stress and anxiety, a future study with a higher study sample can be done to explore the association of IDA with stress and anxiety. Lastly, a reduction in IDA in this age group ensures healthy future pregnancies and lactation.
